# Synthesis and Characterization of Iron Bispyridine Bisdicyanamide, Fe[C_5_H_5_N]_2_[N(CN)_2_]_2_

**DOI:** 10.3390/molecules28134886

**Published:** 2023-06-21

**Authors:** Laura Henrich, Peter C. Müller, Jan Hempelmann, Markus Mann, Jan van Leusen, Simon Steinberg, Richard Dronskowski

**Affiliations:** 1 Chair of Solid State and Quantum Chemistry, Institute of Inorganic Chemistry, RWTH Aachen University, 52056 Aachen, Germany; 2 Institute of Energy and Climate Research (IEK-1) Materials Synthesis and Processing, Forschungszentrum Jülich GmbH, Wilhelm-Johnen-Straße, 52425 Jülich, Germany; 3 Hoffmann Institute of Advanced Materials, Shenzhen Polytechnic, 7098 Liuxian Blvd, Nanshan District, Shenzhen 518055, China

**Keywords:** crystal structure, iron, pyridine, dicyanamide, quantum chemistry, magnetism

## Abstract

Fe[C_5_H_5_N]_2_[N(CN)_2_]_2_ (**1**) was synthesized from a reaction of stoichiometric amounts of NaN(CN)_2_ and FeCl_2_·4H_2_O in a methanol/pyridine solution. Single-crystal and powder diffraction show that **1** crystallizes in the monoclinic space group *I*2/*m* (no. 12), different from Mn[C_5_H_5_N]_2_[N(CN)_2_]_2_ (*P*2_1_/*c*, no. 14) due to tilted pyridine rings, with *a* = 7.453(7) Å, *b* = 13.167(13) Å, *c* = 8.522(6) Å, *β* = 114.98(6)° and *Z* = 2. ATR-IR, AAS, and CHN measurements confirm the presence of dicyanamide and pyridine. Thermogravimetric analysis shows that π-stacking interactions of the pyridine rings play an important role in structural stabilization. Based on DFT-optimized structures, a chemical bonding analysis was performed using a local-orbital framework by projection from a plane-wave basis. The resulting bond orders and atomic charges are in good agreement with the expectations based on the structure analysis. SQUID magnetic susceptibility measurements show a high-spin state Fe^II^ compound with predominantly antiferromagnetic exchange interactions at lower temperatures.

## 1. Introduction

Solid-state materials containing pseudo elements, such as cyanide [1,2,3,4,5,6,7,8], carbodiimide, and cyanamide [9,10,11,12,13,14,15,16,17,18] have gained a lot of interest over the last decades, in particular because those complex anions allow to mimic halide or chalcogenide chemistry with much more covalent nitrogen atoms. Another interesting pseudo-halide entity, the dicyanamide anion (dca), has comparatively gained less attention but is considered important because of its structural behavior and boomerang-shaped appearance. One of its outstanding properties is the ability to stabilize high-spin states due to the weaker ligand field in comparison to cyanide. In addition, the dicyanamide anion can coordinate up to four cations, due to the free electron pairs and the negative charge at the nitrogen atoms. Therefore, dca may act as a bridging ligand between metals [19,20,21,22,23,24,25].

Three-dimensional metal dicyanamides can occur in the so called α- and β-structures. In the α-structure, all nitrogen atoms of the dca moiety are *μ*-1,3,5 bridging ligands, and the metal atom is octahedrally surrounded by nitrogen atoms of six dca units. This results in a rutile-like 3*D* network. In the β-structure, only the terminal nitrogen atoms are coordinated to the metal (*μ*-1,5 bridging), resulting in the metal adopting a tetrahedral coordination of four dca units, thereby forming a layered structure. Due to the boomerang shape of the dca units, the layers are zigzag-shaped. The α-structure is known for 3*d* metals in oxidation states not in favor of Jahn–Teller distortion (e.g., Mn [26,27], Fe, Co, Ni [28]) [29]. In addition to Cu(dca) [30], a β-structure is also known for Co(dca)_2_ [31] and Zn(dca)_2_ [32]. Due to the rutile-like network, 3*d* metal dicyanamides in the α-structure exhibit cooperative magnetism making them interesting research targets [27,28,29]. On the other hand, dicyanamides crystallizing in the β-structure feature free accessibility to the metal as a consequence of their layered structure, a property of potential use in heterogeneous catalysis [23,24,25,26,27,28,29,30,31,32].

By introducing neutrally charged, non-bridging ligands *L* (e.g., DMF, EtOH, pyridine [33,34], pyridazine [35,36], and ammonia [37,38]) to these 3D metal dicyanamides, both structure and properties change to a structure type between the α- and the β-structures. In the latter structure type, only the terminal nitrogen atoms of the dca units are *μ*-1,5 bridging the metals, as said before, so the additional *L* ligands complete the octahedral metal coordination. Independent from the ligand, the dca units and metals can be treated as 2*D* layers built up from interacting 1*D* chains, analogous to the β-structure of pure 3*d* metal dicyanamide compounds. Due to the additional ligands, the different layers or chains can interact with each other leading, for example, to the formation of hydrogen bonds or π-stacking stabilizing the compound. These in-between structures exhibit paramagnetic behavior instead of cooperative magnetism [33,34,35,36,37,38] probably due to the longer exchange pathway of *μ*-1,5 instead of *μ*-1,3,5 dicyanamide anions present in pure 3D metal dicyanamides [36]. In this paper, we discuss the synthesis and characterization of a new *ML*_2_[N(CN)_2_]_2_ compound: Fe[C_5_H_5_N]_2_[N(CN)_2_]_2_.

## 2. Results and Discussion

### 2.1. Structural Description and Discussion

The crystal structure of Fe[C_5_H_5_N]_2_[N(CN)_2_]_2_ was determined and confirmed based on several X-ray diffraction experiments at ambient temperature (300 K) as well as at 100 K. Thorough inspections of these data pointed to the monoclinic system (*a* = 7.453(7) Å, *b* = 13.167(13) Å, *c* = 8.522(6) Å, and *β* = 114.98(6)°), while the structure was solved and refined in space group *I*2/*m* (no. 12). In this context, it should be noted that the previously reported Mn[C_5_H_5_N]_2_[N(CN)_2_]_2_ crystallizes in the space group *P*2_1_/*c* (no. 14) [33]. Therefore, the powder as well as single-crystal X-ray diffraction data were carefully examined in order to confirm the choice of an *I*-centered monoclinic lattice for the iron-containing species (see below and Section 3 for a full discussion regarding the structure determination).

**Figure 1:**
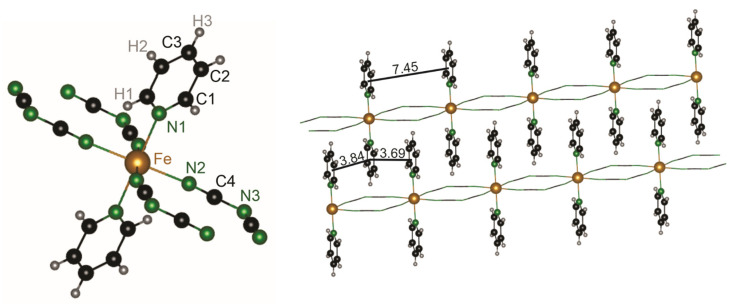
Coordination sphere of the iron atoms in Fe[C_5_H_5_N]_2_[N(CN)_2_]_2_ (**left**) and intercalation of pyridine rings within different chains (**right**). For clarity, the atoms of the dca units were removed in the right subfigure. Fe in gold, N in green, C in black, H in grey.

Each unit cell comprises two formula units, while the bond lengths of the dca units in the iron-containing compound are consistent with data provided for such units in the literature [33,38]. In the dca unit, the distance of the outer C–N bond is *d*(N2–C4) = 1.150(7) Å, while the length of the inner C–N bond is *d*(C4–N3) = 1.308(6) Å. Such C–N distances are indicative of an inner single bond and an outer triple bond, in harmony with a simple Lewis sketch. The angles of the dca units are ∠(N2–C4–N3) = 174.7(5)° and ∠(C4–N3–C4) = 119.5(6)° which are also in good agreement with data in the literature [33,38]. The iron atoms are coordinated by four equatorially arranged dca units and additionally by the nitrogen atoms of two pyridine rings located in *trans* position (Figure 1, left). As a consequence, the coordinating nitrogen atoms form an almost ideal octahedron with distances of *d*(Fe–N1)_pyridine_ = 2.192(11) Å and *d*(Fe–N2)_dca_ = 2.166(5) Å in analogy to the manganese-containing compound reported in the literature (*d*(Mn–N)_pyridine_ = 2.271(3) Å, *d*(Mn–N)_dca_ = 2.226(3) Å). Unlike 3*d* metal dicyanamides, these compounds show *μ*-1,5 bridging in which the central nitrogen atom of the dca unit is not bonded to the metal. Indeed, the dicyanamides form bridging ligands, which connect the iron atoms within 1*D* chains. The pyridine rings of the different chains are intercalated with each other (Figure 1, right).

This results in the formation of 2*D* layers in which the pyridine rings play an important role in stabilizing the structure. A view along the *a*-axis reveals the almost perpendicular N–Fe–N angle of ∠(N2–Fe–N2) = 91.6(3)°. Looking along the *b*-axis, another structural peculiarity becomes apparent. The pyridine rings of different 2*D*-layers are ordered in a parallel manner. Notably, the pyridine rings in the manganese-containing compound are tilted by circa 17° individually, alternating every second layer [33]. This circumstance explains the cancellation of translation symmetry, and therefore the symmetry reduction of the manganese-containing compound in comparison to the iron-containing species. The distances between the intercalated rings alternate between 3.844(17) Å and 3.692(18) Å, which is well within the range of a typical π-stacking distance [39,40]. Seemingly, this stacking additionally stabilizes the substance that is neither sensitive to air nor moisture, even insoluble in water or selected organic solvents. 

**Figure 2:**
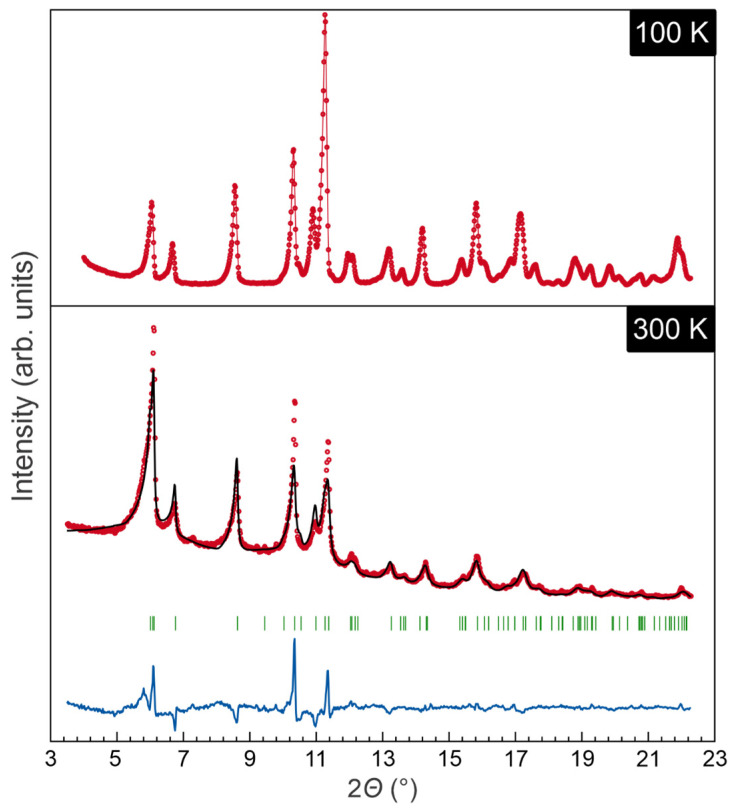
Comparison of the X-ray reflection-geometry measurement at 100 K (**top**) and the Rietveld refinement of the transition-geometry measurement at 300 K (**bottom**) with measured data in red, fitted pattern in black, Bragg positions in green, and difference curve in blue.

Because the anisotropic atomic displacement parameters (ADPs) corresponding to certain atoms of the pyridine rings are evidently elongated along the *c* direction, we also probed if the crystal structure of the iron-containing species could undergo a structural transition to that of the manganese-containing compound. Therefore, additional powder X-ray diffraction experiments and Rietveld refinements of the recorded data were carried out at different temperatures. The results of the refined lattice parameters at 300 K (Figure 2, Table S1) is in good agreement with those obtained in the single-crystal X-ray diffraction experiments (*R*_B_ = 4.67%), while the 100 K measurement could not be refined due to the X-ray diffraction mode chosen (reflection, not transmission). Nonetheless, the reflection data show that all reflections could be described with the theoretical pattern generated by the single-crystal measurement, the deficiencies showing up in the profiles. Further Rietveld refinements based on the structure model of the manganese-containing compound did not yield a reasonable model. We therefore conclude that the aforementioned structural transition is not evident for the iron-containing species between 300 and 100 K, possibly at even lower temperatures. Based on the single-crystal X-ray diffraction data, we checked if a modulated structure model might show up from a temperature-dependent rattling of the pyridine rings around the N1–C3–H3 axis; there was also no clear evidence of a modulated structure model. Therefore, further research including high-quality neutron diffraction experiments within a broader temperature range might be needed to possibly monitor a movement of the pyridine rings.

### 2.2. Elemental Analysis

The atomic absorption spectroscopy (AAS) and CHN measurement show the presence of iron, carbon, nitrogen, and hydrogen (Table 1). The value for iron (from AAS) is slightly larger, and those of carbon, hydrogen, and nitrogen are slightly smaller than expected. A plausible explanation for this can be the presence of other ions bound in the sample, causing the carbon, hydrogen, and nitrogen values to decrease. For example, the surface of the compound could still be contaminated by chloride ions originating from FeCl_2_ used during synthesis. Since the compound is a microcrystalline powder, the surface is relatively large and could therefore influence the overall composition.

### 2.3. Attenuated Total Reflection Infrared Spectroscopy (ATR-IR)

The obtained IR frequencies show the presence of both the dca unit and the pyridine ring (Figure S1), which is in good agreement with the literature [21,36]. For a better comparison, the theoretical vibrations of Fe[C_5_H_5_N]_2_[N(CN)_2_]_2_, Fe[N(CN)_2_]_2_ and pyridine were distinguished based on DFT and they show a good fit with the experimental data. The assignments of the vibrations can be found in Table S5.

### 2.4. Thermogravimetric Analysis (TGA)

**Figure 3:**
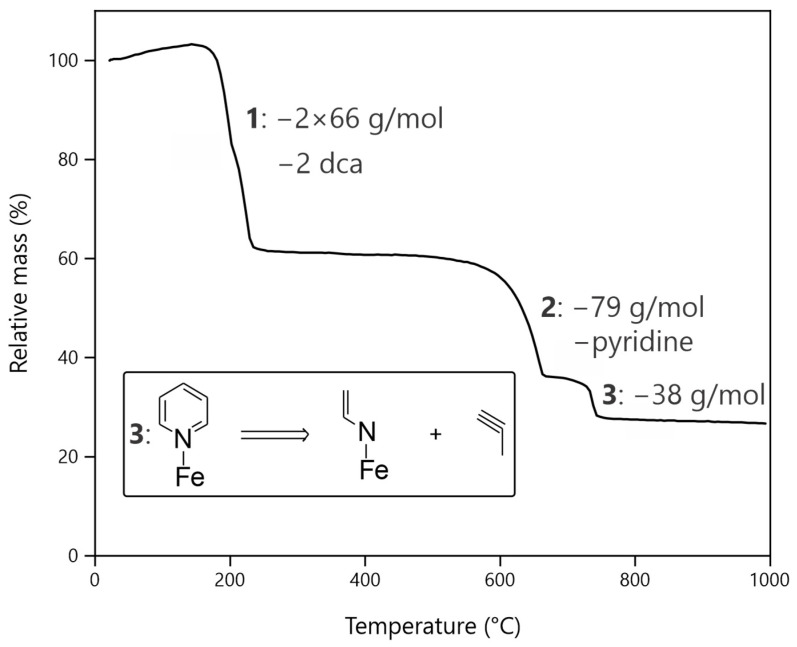
Thermogravimetric analysis of Fe[C_5_H_5_N]_2_[N(CN)_2_]_2_ measured in nitrogen flow; inset: possible decomposing reaction in step 3.

The TGA data are depicted as a mass fraction vs. temperature plot in Figure 3.

Mass losses occur in three distinguishable steps at approx. 180 (**1**), 620 (**2**), and 670 °C (**3**). Assuming that the substance is the desired compound (Fe[C_5_H_5_N]_2_[N(CN)_2_]_2_), the first mass loss (**1**) of 100 to 61% fits the cleavage of two dca units (132.08 g/mol), leaving Fe[C_5_H_5_N]_2_ behind. The mass loss at 620 °C (**2**) can be explained by the cleavage of one pyridine ring per iron atom (79.10 g/mol). The mass loss is somewhat surprising at such a high temperature, since the boiling point of pyridine is at 115 °C under standard conditions. Hence, significant covalency in the bond between iron and pyridine does exist, and π-stacking also exists between pyridine rings. According to the literature, the cleavage temperature of pyridine is about 400 °C [41], which is almost 200 °C below the temperature needed for splitting a pyridine ring from the compound. The last decomposition step at 670 °C (**3**) fits a mass loss of 38.06 g/mol and can therefore not be explained by the cleavage of another pyridine ring. Comparison with the literature indicates that pyridine may decompose into many different components although there is no decomposition fitting a mass loss of about 38 g/mol, while leaving a reasonable iron residue [42,43]. The high temperature may have caused the ring to split, resulting in the cleavage of a C_3_H_3_ fragment (Figure 3, inset). These fragments could have reacted with each other after cleavage to saturate the free-binding sites on the carbon. Such a reaction was not tracked in the TGA but seems the most reasonable to us.

In summary, TGA confirms the presence of the proposed fragments in the compound. Furthermore, the interactions of the pyridine rings have a very large influence on the stability of the compound, which is already reflected in its low solubility.

### 2.5. Chemical Bonding Analysis

**Figure 4:**
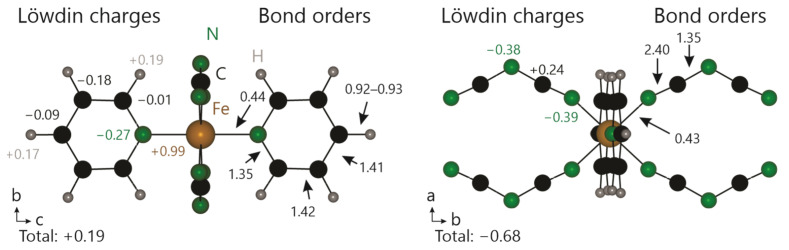
ICOBIs and Löwdin charges of Fe[C_5_H_5_N]_2_[N(CN)_2_]_2_; (**left**): in *a* direction, (**right**): in *c* direction.

To examine the chemical bonding situation in Fe[C_5_H_5_N]_2_[N(CN)_2_]_2_, the crystal orbital bond index (COBI) [44] as well as Löwdin charges [45] were calculated using LOBSTER. In contrast to common bonding indicators for solids, COBI’s interpretation is almost trivial as its energy integral, the ICOBI, directly translates into the covalent bond order between any two atoms. ICOBI as well as the charges for all respective atoms are shown in Figure 4.

All calculated bonding descriptors are in good agreement with expectations from a simple Lewis picture: both C–C and C–N bonds in the pyridine rings have an ICOBI of about 1.4, indicative of the aromatic ring. The C–H bonds are single bonds, as expressed by ICOBI ≈ 0.92. With an overall charge of +0.19, pyridine may be considered almost neutral. The dicyanamide unit shows a bond order of 2.4 between nitrile nitrogen and carbon, while the bond order between amide nitrogen and carbon is 1.35. In combination, both bond orders indicate the presence of mesomerism between the N≡C–N and N=C=N forms of the dicyanamide molecule. The calculated bond orders in the dca unit match those in the proposed structure of Fe[C_5_H_5_N]_2_[N(CN)_2_]_2_. The charge on the formally divalent iron atom indirectly suggests a strong covalent moiety in the coordinative bonds between iron and nitrogen, confirmed by the ICOBIs of 0.43–0.44 for the Fe–N bonds.

### 2.6. Magnetic Properties

**Figure 5:**
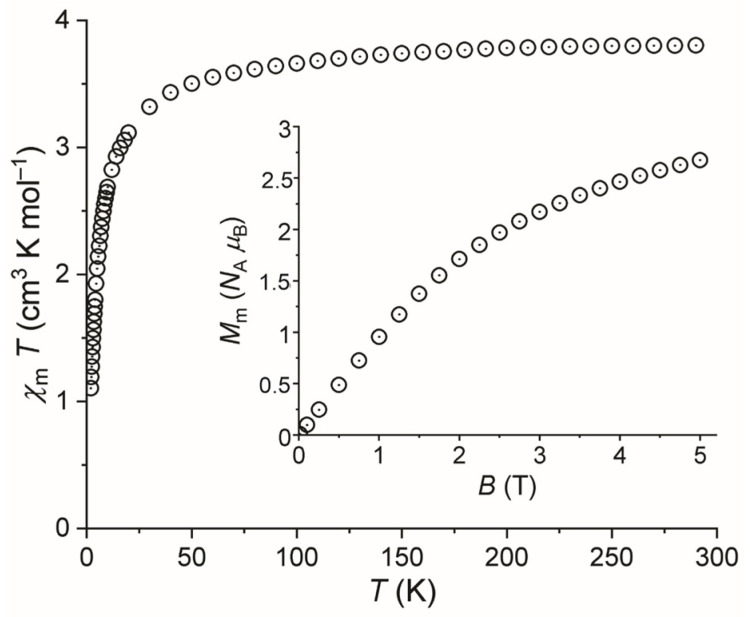
Temperature dependence of *χ*_m_*T* of Fe[C_5_H_5_N]_2_[N(CN)_2_]_2_ at 0.1 T; inset: molar magnetization *M*_m_ vs. applied magnetic field *B* at 2.0 K.

The magnetic data of Fe[C_5_H_5_N]_2_[N(CN)_2_]_2_ normalized to a single Fe^II^ center are displayed in Figure 5 as *χ*_m_*T* vs. *T* plot at 0.1 T and as *M*_m_ vs. *B* plot at 2.0 K (inset).

At 290 K, *χ*_m_*T* is 3.80 cm^3^ K mol^−1^, which is within the expected [46] range of 3.2–4.1 cm^3^ K mol^−1^ of an isolated high spin Fe^II^ center. This range is above the spin-only value of 3.00 cm^3^ K mol^−1^ (for *S* = 2), since orbital contributions are not fully quenched for an octahedrally coordinated 3*d*^6^ ion. Upon cooling the compound, the values of *χ*_m_*T* continuously decrease and drop off at temperatures below 50 K, reaching 1.11 cm^3^ K mol^−1^ at 2 K. At this temperature, the molar magnetization is an almost linear function of the applied magnetic field up to about 1.5 T. Upon increasing fields, *M*_m_ increases at a decreasing rate without reaching saturation at the highest field of the experimental set-up of 5.0 T. The value of *M*_m_ is 2.7 *N*_A_ *μ*_B_, which is well below the saturation value of about 4.5 *N*_A_ *μ*_B_ estimated from the value of *χ*_m_*T* at 290 K. The significant decrease in *χ*_m_*T* at temperatures below 100 K is partly due to the thermal depopulation of the energy states of the Fe^II^ center split by electron–electron inter-repulsion, ligand field, and spin–orbit coupling. However, the main contribution is due to predominantly antiferromagnetic, yet weak exchange interaction between neighboring Fe^II^ centers. This is also evident from the *M*_m_ vs. *B* curve. On the one hand, the magnetization of an isolated high spin Fe^II^ center with distorted octahedral symmetry is not expected to reach saturation at 5 T and 2 K due to the ground state being characterized by distinct mixing of energy states with different magnetic moments. In addition to the latter, such Fe^II^ centers are additionally characterized by magnetic anisotropy that yields lower values of *M*_m_ since they represent the mean values obtained from a sample of randomly oriented crystallites, i.e., a powdered material. However, both effects result in a value of *M*_m_ of about 1 *N*_A_ *μ*_B_ below the saturation value and a slightly smaller slope at 5 T and 2 K than shown in Figure 5. Therefore, the even lower value of *M*_m_ at this temperature and field also reveals weak, predominantly antiferromagnetic exchange interactions within the compound. This observation fits well with the fact that the corresponding manganese compound Mn[C_5_H_5_N]_2_[N(CN)_2_]_2_ has also been described as being an antiferromagnet, with an effective moment around 5.85 *μ*_B_ at 300 K and weak antiferromagnetic intrachain interactions mediated by the Mn[N(CN)_2_]_2_Mn pathways [33].

## 3. Materials and Methods

### 3.1. Synthesis

Fe[C_5_H_5_N]_2_[N(CN)_2_]_2_ was synthesized by adding FeCl_2_·4 H_2_O (0.9942 g, 5 mmol) to a stirring solution of NaN(CN)_2_ (0.8904 g, 10 mmol, 2 Eq.) in 100 mL methanol. Afterwards, pyridine (1.5 mL, ≈18.5 mmol, 2.9 Eq.) was added dropwise. The beige-colored product precipitated immediately, was filtered, dried, and appeared as a crystalline powder containing a few single crystals, which were stable against air and moisture.

### 3.2. Single-Crystal Diffraction

The samples were selected from the bulk and placed at the top of glass fibers with the aid of grease. The sets of single-crystal X-ray intensity data were collected at 100 K on a Bruker^®^ APEX CCD diffractometer (Bruker Inc., Madison, WI; Mo-K_α1_ radiation, *λ* = 0.71073 Å) that was equipped with an Oxford^®^ Cryostream 700 to control the temperature of the measurements. Initial indexing of the collected data pointed to a *C*-centered monoclinic lattice setting that was used for subsequent processing of the data. The raw intensity data were integrated using the program *SAINT* [47], while an absorption correction was accomplished utilizing the *SADABS* code [48]. Applications of the reflection conditions to the data sets were accomplished via the *XPREP* algorithms within the *APEX II* suite [49] and pointed to the space group *I*2/*m*, which was also used for the final structure solutions and refinements. The crystal structure was solved using direct methods (*SHELXS-97*) and refined in full-matrix least squares on *F*^2^ (*SHELXL-2014*) which also included anisotropic atomic displacement parameters [50,51]. In the framework of the data analysis and structure solution, we became aware of reflections of very weak intensities which translated into the large *R*_1_ value for all data, while somewhat enlarged anisotropic atomic displacement parameters were evident for certain sites assigned to the pyridine rings. Therefore, extensive examinations were undertaken to check for the presence of a space group being different from *I*2/*m* or for the occurrence of a modulated structure as a consequence of possible elongations of the pyridine rings; yet, none of our attempts could satisfactorily reveal the existence of one of the aforementioned phenomena, while close inspections of the intensity data sets did not indicate the presence of any sort of twinning. In addition, we probed the presence of positional disorder for the C1 and C2 sites of the pyridine ligands. These refinement cycles resulted in notable distortions of the pyridine rings, however, the ligand *C*_2v_ symmetry was entirely lost, even though substantial improvements of the refinements were not accomplished. Because there is no effect which could reasonably provoke such a distortion of the pyridine rings, positional disorder of the C1 and C2 positions was discarded. Furthermore, we took into consideration to carry out a numerical absorption correction; yet, the size of the selected single crystal (≤0.1 mm) hindered to conduct a numerical absorption correction and hence a semi-empirical method had to be used in line with the recommendations of the International Union of Crystallography [52].

### 3.3. Powder X-ray Diffraction

The powder sample was first loaded into a sample holder and then transferred to a Stoe^®^ STADI MP diffractometer (Stoe & Cie, Darmstadt, Germany; Mo-K_α1_ radiation, (λ = 0.71073 Å), which was used to collect the powder X-ray diffraction data set in transmission mode at 300 K, while the 100 K measurement was performed in reflection mode under vacuum. The measurements were controlled via the WinXPow^®^ program [53], which was also used for further processing of the raw data, while the Rietveld refinements of the collected powder X-ray diffraction patterns for the measurement at *T* = 300 K were performed with *FullProf Suite* [54] and a formally pseudo-Voigt profile function of full Lorentzian shape to profit from the better asymmetry correction. The atomic positions and ADPs were taken from the single crystal refinement and were not refined further. The results of this Rietveld refinement further confirmed the structural model determined on the basis of the single-crystal X-ray diffraction experiments (see above).

### 3.4. Magnetic Measurements

A polycrystalline sample of Fe[C_5_H_5_N]_2_[N(CN)_2_]_2_ was compacted and immobilized into a cylindrical PTFE capsule. A Quantum Design^®^ MPMS-5XL SQUID magnetometer (Quantum Design, San Diego, CA, USA) was employed to collect the magnetic data, which were acquired as a function of temperature (2–290 K at 0.1 T) and magnetic field (0.1−5 T at 2 K). The data were corrected for the diamagnetic contributions of the sample holder and the compound (*χ*_m,dia_ = −1.73 × 10^−4^ cm^3^ mol^−1^).

### 3.5. Infrared Spectroscopy (ATR-IR)

Fine powders of Fe[C_5_H_5_N]_2_[N(CN)_2_]_2_ were loaded in a sample holder, while the IR spectra (absorbance versus wavelength) were collected on a Bruker^®^ alpha II spectrometer (Bruker, Madison, WI, USA). The program OPUS was used for the controlling the measurements as well as for processing the raw data.

### 3.6. Thermogravimetric Analysis (TGA)

Thermogravimetric analyses were performed using Netzsch^®^ STA 409 C (Netzsch, Selb, Germany). The compound was heated and weighed in nitrogen flow in the temperature range from 25 to 1000 °C with a heating rate of 5 °C/min.

### 3.7. Atomic Absorption Spectroscopy (AAS)

For the determination of the iron content, approx. 50 mg of each sample were dissolved three times in 5 mL concentrated HCl and quantitatively transferred to a 100 mL volumetric flask. To each sample, 10 mL of a 0.1% KCl solution was added as an ionization buffer, and the volumetric flasks were filled up to the calibration mark. The sample solutions were analyzed using a novAA300 AAS from Analytik Jena, Germany; the reference solutions were prepared from a certified iron standard (1000 mg/L) from Kraft. A six-element multi-HKL from L.O.T. Oriel served as the emission source.

### 3.8. Computational Details

All structures were optimized via density functional theory (DFT) using the *Vienna* Ab initio *Simulation Package* (VASP) [55,56,57,58]. The electronic wave functions were modeled with PAW pseudopotentials [59] with a kinetic energy plane-wave cutoff of 500 eV. The exchange–correlation interactions were modeled using the generalized gradient approximation (GGA) as parametrized by Perdew, Burke and Ernzerhof and optimized for solids (PBEsol) [60] with an additional D3-correction term introduced by Grimme with Becke–Johnson damping [61,62]. The Brillouin-zone integration was done using Blöchl’s tetrahedron method, employing **k**-point meshes with densities between 0.02 and 0.04 Å^−1^ [63]. Convergence of the calculations was assumed for energy differences of 10^−4^ eV for ionic steps and 10^−6^ eV for electronic steps.

After the structural optimization, a chemical bonding analysis was performed. Because the bonding analysis required the use of local orbitals whose nature is in stark contrast to that of the plane waves, the electronic ground-state wave functions were unitarily transformed onto a local-orbital basis using the *Local Orbital Basis Suite Towards Electronic-Structure Reconstruction* (LOBSTER) package [64,65,66]. The simulation of the lattice vibration was performed using the program *Phonopy* [67] according to the Hellmann–Feynman forces from VASP. A simulation of IR spectra was conducted based on phononic calculations and Born effective charges received using the methods described above and processed using the *JaGeo*/IR software package [68,69,70,71]. 

## 4. Conclusions

Herein, we report the synthesis and characterization of iron bispyridine bisdicyanamide, Fe[C_5_H_5_N]_2_[N(CN)_2_]_2_, which is a new transition metal-containing compound with dca and pyridine ligands. The crystal structure was determined using single-crystal as well as powder X-ray diffraction with particular regard to a possible motion of the pyridine rings; yet, a structural transition related to a reorientation of the pyridine rings could not be encountered based on X-ray diffraction. AAS, CHN, ATR-IR, and TGA measurements were performed to prove the composition. Additionally, the TGA indicates the high impact of the π-stacking on the structural stability. The bonding situation in the compound, examined via ICOBI and Löwdin charge analyses, agrees with the expectations based on structural analysis. Magnetic measurements evidence that Fe[C_5_H_5_N]_2_[N(CN)_2_]_2_ is a paramagnetic compound comprising high spin Fe^II^ centers. There are weak, predominantly antiferromagnetic exchange interactions between these atoms, observable at low temperatures. Considering the structural information, these interactions are most likely of 1*D* nature between the Fe^II^ centers coupled through the dca ligands, i.e., along the Fe–(dca)_2_–Fe–chains within the compound. 

## Figures and Tables

**Table 1:** Theoretical and experimental data for Fe[C_5_H_5_N]_2_[N(CN)_2_]_2_ of the AAS (iron) and CHN (carbon, hydrogen, and nitrogen) measurement.

Element	Fe	C	H	N
Theoretical (%)	16.13	48.58	2.91	32.37
Experimental (%)	17.3(2)	45.9(1)	2.60(8)	31.4(2)
Deviation (abs.)	+1.17	−2.73	−0.30	−1.02

## Data Availability

CCDC 2265156 contain the crystallographic data for this paper. These data can be obtained free of charge via http://www.ccdc.cam.ac.uk/conts/retrieving.html or from the CCDC, 12 Union Road, Cambridge CB21EZ; Fax: +44-1223-336033; E-Mail: deposit.ccdc.cam.ac.uk. All other data may be obtained from the corresponding author on reasonable request.

## Supplementary Materials

Single-crystal data in CIF format can be downloaded as supporting information at: https://www.mdpi.com/article/10.3390/molecules28134886/s1. Table S1: Data of the single crystal refinement of Fe[C_5_H_5_N]_2_[N(CN)_2_]_2_; Table S2: Data of Rietveld refinement of powderous [C_5_H_5_N]_2_[N(CN)_2_]_2_ sample shown in Figure 2; Table S3: Atomic positions from single crystal refinement; Table S4: Anisotropic displacement parameters for iron, carbon and nitrogen and isotropic displacement parameters for hydrogen in **1** from single crystal refinement; Figure S1: ATR-IR measurement of **1** between 4000 and 400 cm^–1^; Table S5: ATR-IR for **1** with all assigned vibrations below 2500 cm^–1^.

## Sample Availability

Samples of the compound **1** are available from the authors.
