# Supplementary material for: Synthesis and Characterization of Iron Bispyridine Bisdicyanamide, Fe[C5H5N]2[N(CN)2]2
Source: Molecules. 2023 Jun 21;28(13):4886. doi: 10.3390/molecules28134886 (PMC10343607; doi:10.3390/molecules28134886)
Supplement: Supplementary file 1 [file molecules-28-04886-s001.zip › molecules-2443465-supplementary.pdf]

Supplementary Materials

Synthesis and Characterization of Iron Bispyridine Bisdicyanamide, $Fe[C_5H_5N]_2[N(CN)_2]_2$

Laura Henrich [1], Peter C. Müller [1], Jan Hempelmann [1], Markus Mann [1,2], Jan van Leusen [1], Simon Steinberg [1] and Richard Dronskowski [1,3,*]

[1] Chair of Solid State and Quantum Chemistry, Institute of Inorganic Chemistry, RWTH Aachen University, 52056 Aachen, Germany. Fax: +49 241 80-92642; Tel: +49 241 80-93642
[2] Institute of Energy and Climate Research (IEK-1) Materials Synthesis and Processing, Forschungszentrum Jülich GmbH, Wilhelm-Johnen-Straße, 52425 Jülich, Germany
[3] Hoffmann Institute of Advanced Materials, Shenzhen Polytechnic, 7098 Liuxian Blvd, Nanshan District, Shenzhen, China
* Correspondence: drons@HAL9000.ac.rwth-aachen.de

Table S1. Data of the single crystal refinement of $Fe[C_5H_5N]_2[N(CN)_2]_2$.

Chemical formula	$FeC_{14}N_8H_{10}$
Formula weight (g/mol)	346.15
Crystal system	monoclinic
Space group (no.)	*I*2/*m* (12)
Temperature (K)	100(2)
a (Å)	7.453(7)
b (Å)	13.167(13)
c (Å)	8.522(6)
β (°)	114.98(6)
V (Å^3)	758.1(12)
Z	2
Crystal shape and color	brown-beige block
ρ_{calc} (g·cm^{-3})	1.516
Radiation, λ (Å)	Mo-Kα_1, 0.71073
Diffractometer	Bruker APEX CCD
Absorption correction	Multi-scan, SADABS 2014/5
T_{min}, T_{max}	0.4236, 0.7461
No. of measured, independent, and observed [I>2σ(I)] reflections	5814, 1159, 676
R_1	15.29%
R_{1obs}	7.42%
R_{all}	17.08%
R_{1all}	14.45%
GOF_{obs}	1.057
No. of parameters, restraint, constraints	58, 0, 3
H-atom treatment	Constrained refinement

Table S2. Data of Rietveld Refinement of powderous $[C_5H_5N]_2[N(CN)_2]_2$ sample shown in Figure 2.

Chemical formula	$FeC_{14}N_8H_{10}$	ρ_{calc} (g·cm^{-3})	1.479
Formula weight	346.15	Radiation, λ (Å)	Mo-Kα_1, 0.71073
Crystal System	monoclinic	Profile function	pseudo-Voigt w. axial divergence asymmetry
Space group (No.)	*I* 2/*m* (12)	*U*	5.616(38)
Temperature (K)	300	*V*	–0.675(16)
a (Å)	7.437(99)	*W*	0.022(38)
b (Å)	13.300(82)	S_L/S_D	0.027(3)
c (Å)	8.652(34)	η	1.0
β (°)	114.68(1)	R_P (%)	4.34
V (Å^3)	777.36(17)	R_{wp} (%)	2.16
Z	2	X^2	12.9

Table S3. Atomic positions from single crystal refinement.

**Atom**	**Site**	***x***	***y***	***z***
Fe1	2*a*	0	0	0
N1	4*i*	0.1390(6)	0	0.2837(6)
N2	8*j*	0.2083(5)	0.1147(3)	0.9998(4)
N3	4*h*	½	0.2015(4)	0
C1	8*j*	0.1869(10)	0.0842(4)	0.3740(6)
C2	8*j*	0.2840(11)	0.0866(5)	0.5525(7)
C3	4*i*	0.3363(9)	0	0.6439(8)
C4	8*j*	0.3483(6)	0.1515(3)	0.9998(5)
H1	8*j*	0.1530	0.1470	0.3136
H2	8*j*	0.3141	0.1500	0.6110
H3	4*i*	0.4063	0	0.7665

Table S4. Anisotropic displacement parameters for iron, carbon and nitrogen and isotropic displacement parameters for hydrogen in **1** from single crystal refinement.

**Atom**	U_{11}	U_{22}	U_{33}	U_{12}	U_{13}	U_{23}
Fe1	0.0145(6)	0.0243(7)	0.0210(7)	0	0.0090(5)	0
N1	0.018(2)	0.024(3)	0.024(3)	0	0.010(2)	0
N2	0.023(2)	0.029(2)	0.027(2)	0.0057(15)	0.0097(17)	0.0016(15)
N3	0.029(3)	0.012(3)	0.069(4)	0	0.033(3)	0
C1	0.114(5)	0.030(3)	0.023(3)	–0.003(2)	–0.004(3)	0.034(3)
C2	0.138(7)	0.037(4)	0.029(3)	–0.011(3)	0.000(3)	0.040(4)
C3	0.023(3)	0.040(4)	0.027(3)	0	0.009(3)	0
C4	0.024(2)	0.016(2)	0.022(2)	0.0011(16)	0.0097(18)	0.0056(16)
			U_{iso}			
H1			0.080			
H2			0.096			
H3			0.037			

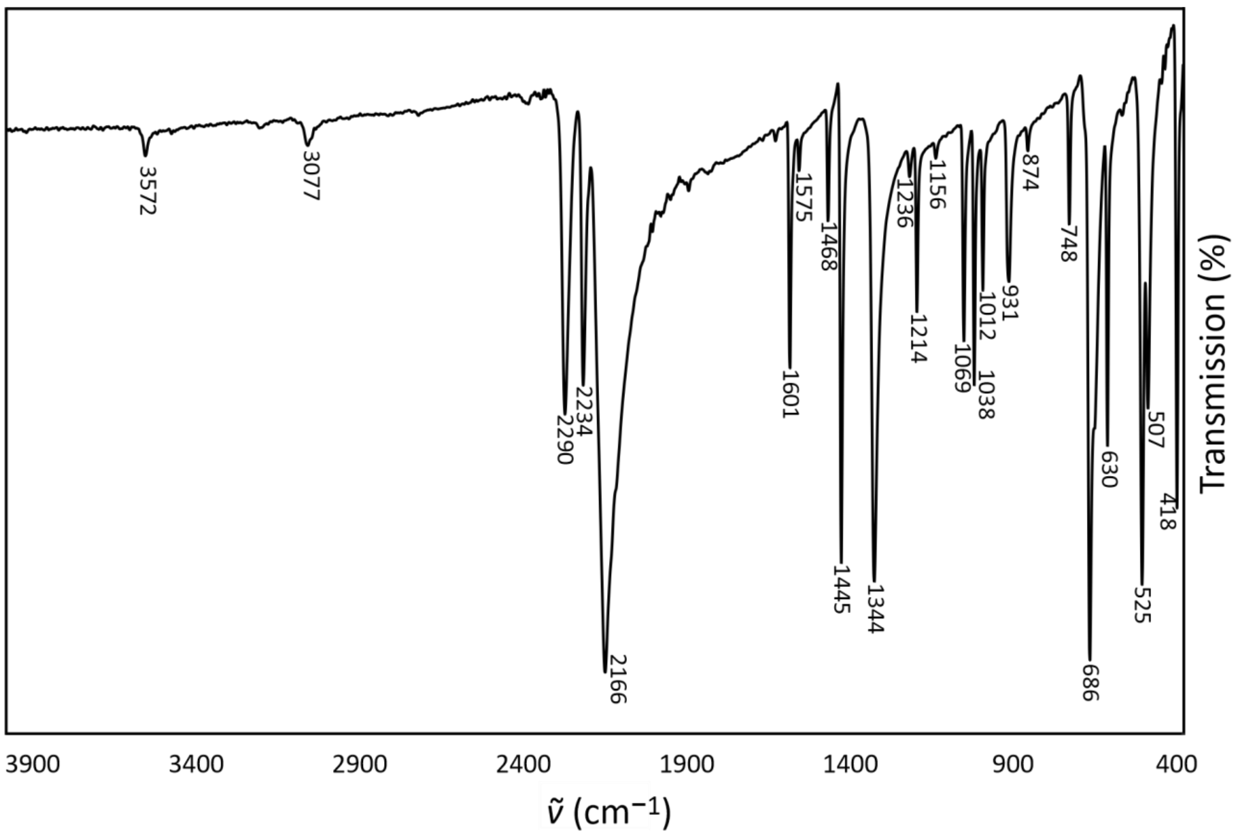

Figure S1. ATR-IR measurement of **1** between 4000 and 400 cm^{-1}.

Table S5. ATR-IR for **1** with all assigned vibrations below 2500 cm^{-1}.

$\tilde{\nu}$ (cm^{-1}) measured	$\tilde{\nu}$ (cm^{-1}) calculated	$\tilde{\nu}$ (cm^{-1}) $Fe[N(CN)_2]_2$ calculated	$\tilde{\nu}$ (cm^{-1}) Pyridine calculated	Vibration
418	420	435	404	γ_s(C–C) + υ(Fe–N) + υ(NC–N–CN)
507/525	490/505	470	-	σ_{as}(N–C≡N)
630/686	625/685	665	-	δ_s(C–C)
748	745	-	702/748	δ_s(C–H)
874*	-	-	-	γ_s(C–C) + υ(Fe–N) + υ(NC–N–CN)
931	925	955	910	ν_s(N–C)
1012	1010	-	986	δ_{as}(C–C)
1038	1035	-	1045	δ_s(C–H)
1069	1070	-	1057	σ_{as}(N–C≡N) + σ_s(N–C≡N)
1154*	-	-	-	δ_s(C–C)
1214	1201	-	1174	δ_{as}(C–H)
1234*	-	-	-	δ(C–C)
1344	1331	1310	-	ν_{as}(N–C)
1445/1469/1601	1435/1465/1596	-	1411/1452/1583	ν_s(C–C)
1574*	-	-		γ_s(C–H)
1647*	-	-	-	ν_s(N–C)
2166*	-	-	-	σ_{as}(N–C≡N) + σ_s(N–C≡N)
2234	-	2275	-	ν_{as}(N–C) + ν_s(N–C)
2290	2239/2280	2305	-	ν_s(N≡C)
2409*	-	-	-	δ_{as}(C–H)

*Overtone, cannot be calculated by DFT.
